# 4′-(4-Bromo­phen­yl)-1′-methyl­dispiro­[acenaphthyl­ene-1,2′-pyrrolidine-3′,2′′-indane]-2,1′′(1*H*)-dione

**DOI:** 10.1107/S1600536812014213

**Published:** 2012-04-13

**Authors:** Ang Chee Wei, Mohamed Ashraf Ali, Tan Soo Choon, Suhana Arshad, Ibrahim Abdul Razak

**Affiliations:** a Institute for Research in Molecular Medicine, Universiti Sains Malaysia, Minden 11800, Penang, Malaysia; b School of Physics, Universiti Sains Malaysia, 11800 USM, Penang, Malaysia

## Abstract

In the title compound, C_30_H_22_BrNO_2_, the cyclo­pentane ring of the dihydro­acenaphthyl­ene group and the pyrrolidine ring are both in envelope conformations with the spiro C atom and N atom, respectively, as the flap atom. The cyclo­pentane ring of the indane group adopts a half-chair conformation. A weak intra­molecular C—H⋯O hydrogen bond forms an *S*(8) ring motif. The naphthalene ring system of the dihydro­acenaphthyl­ene group forms dihedral angles of 41.76 (6) and 42.17 (6)° with the benzene ring of the bromo­phenyl group and the benzene ring of the indane group, respectively. The dihedral angle between the two benzene rings is 83.92 (7)°. In the crystal, mol­ecules are linked by weak C—H⋯O and C—H⋯N hydrogen bonds into a two-dimensional network parallel to the *ac* plane. Weak C—H⋯π inter­actions are also observed.

## Related literature

For related structures, see: Wei, Ali, Ismail *et al.* (2011 ▶); Wei, Ali, Yoon *et al.* (2011 ▶); Wei, Ali, Choon *et al.* (2011 ▶). For ring conformations, see: Cremer & Pople (1975 ▶). For hydrogen-bond motifs, see: Bernstein *et al.* (1995 ▶). For the stability of the temperature controller used in the data collection, see: Cosier & Glazer (1986 ▶).
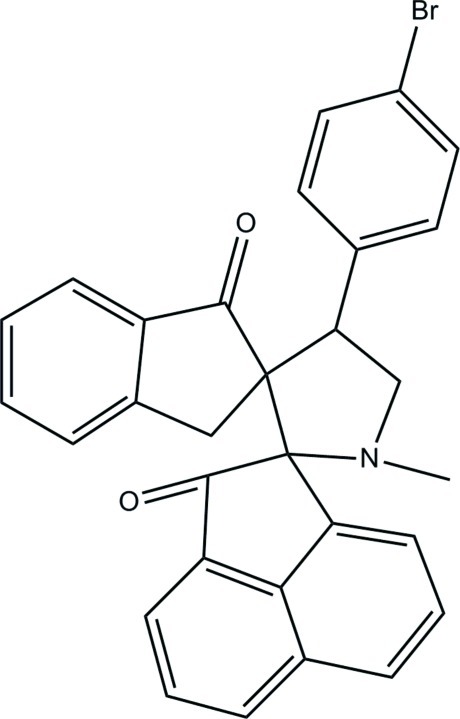


## Experimental

### 

#### Crystal data

C_30_H_22_BrNO_2_
*M* *_r_* = 508.40
Monoclinic, 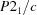

*a* = 8.6638 (1) Å
*b* = 19.9429 (2) Å
*c* = 13.5225 (1) Å
β = 94.937 (1)°
*V* = 2327.77 (4) Å^3^
*Z* = 4
Mo *K*α radiation
μ = 1.80 mm^−1^
*T* = 100 K
0.42 × 0.19 × 0.17 mm

#### Data collection

Bruker SMART APEXII CCD area-detector diffractometer
Absorption correction: multi-scan (*SADABS*; Bruker, 2009 ▶) *T*_min_ = 0.522, *T*_max_ = 0.748
31742 measured reflections
9279 independent reflections
6879 reflections with *I* > 2σ(*I*)
*R*_int_ = 0.034

#### Refinement

*R*[*F* ^2^ > 2σ(*F* ^2^)] = 0.036
*wR*(*F* ^2^) = 0.085
*S* = 1.03
9279 reflections
308 parameters
H-atom parameters constrained
Δρ_max_ = 0.46 e Å^−3^
Δρ_min_ = −0.49 e Å^−3^

### 

Data collection: *APEX2* (Bruker, 2009 ▶); cell refinement: *SAINT* (Bruker, 2009 ▶); data reduction: *SAINT*; program(s) used to solve structure: *SHELXTL* (Sheldrick, 2008 ▶); program(s) used to refine structure: *SHELXTL*; molecular graphics: *SHELXTL*; software used to prepare material for publication: *SHELXTL* and *PLATON* (Spek, 2009 ▶).

## Supplementary Material

Crystal structure: contains datablock(s) global, I. DOI: 10.1107/S1600536812014213/lh5443sup1.cif

Structure factors: contains datablock(s) I. DOI: 10.1107/S1600536812014213/lh5443Isup2.hkl

Additional supplementary materials:  crystallographic information; 3D view; checkCIF report

## Figures and Tables

**Table 1:** Hydrogen-bond geometry (Å, °) *Cg*1 is the centroid of the C15–C20 ring.

*D*—H⋯*A*	*D*—H	H⋯*A*	*D*⋯*A*	*D*—H⋯*A*
C29—H29*A*⋯O1	0.95	2.25	3.1110 (17)	151
C4—H4*A*⋯O2^i^	0.95	2.59	3.3743 (19)	140
C16—H16*A*⋯N1^ii^	0.95	2.50	3.4278 (19)	165
C26—H26*A*⋯O1^iii^	0.95	2.43	3.2597 (18)	146
C5—H5*A*⋯*Cg*1^iv^	0.95	2.71	3.3186 (15)	123

Symmetry codes: (i) 
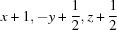
; (ii) 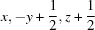; (iii) 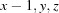; (iv) 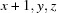.

**Fig. 1.**
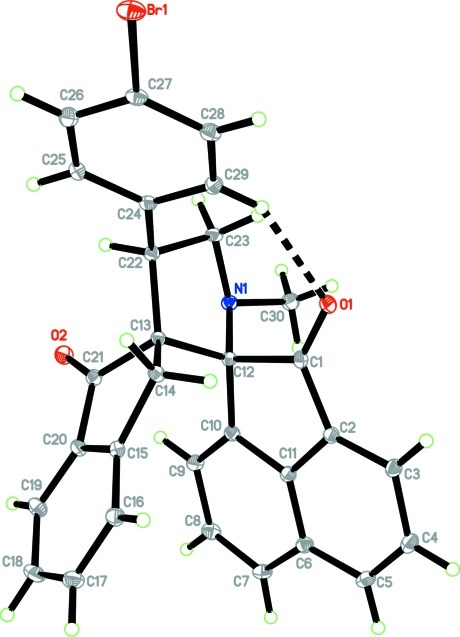
The molecular structure of the title compound, showing 30% probability displacement ellipsoids and the atom-numbering scheme. The dashed line indicates a weak hydrogen bond.

**Fig. 2.**
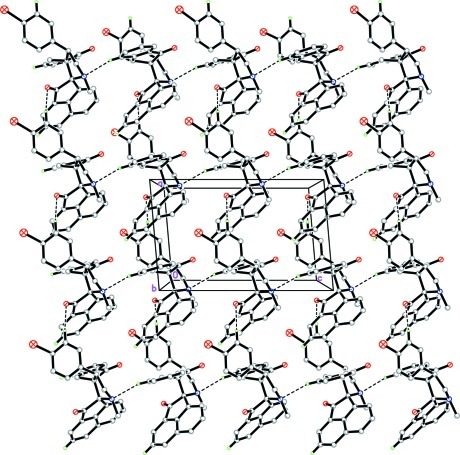
The crystal packing of the title compound. The H atoms not involved in the intermolecular interactions (dashed lines) have been omitted for clarity.

## supplementary crystallographic information

### Comment

As part of our ongoing search to discover novel heterocyclic compounds with antitubercular activity (Wei, Ali, Ismail *et al.*, 2011; Wei, Ali, Yoon *et al.*, 2011), our group has determined the crystal structure of the title compound.

In the molecular structure (Fig 1), the cyclopentane ring within the dihydro-acenaphthylene moiety (C1/C2/C10–C12) [puckering parameters, Q= 0.0742 (15) Å and φ= 151.2 (11)°] and the pyrrolidine ring (N1/C12/C13/C22/C23) [puckering parameters, Q= 0.4112 (14) Å and φ= 185.8 (2)°] are both in envelope conformation (Cremer & Pople, 1975) with atom C12 and atom N1 at the flap, respectively. Meanwhile, the cyclopentane ring (C13–C15/C20/C21) within the dihydro-indene moiety is twisted about C13–C14 bond [puckering parameters, Q= 0.2467 (15) Å and φ= 189.1 (3)°], thereby adopting half-chair conformation. The bond lengths and angles are within normal ranges and comparable to those related structures (Wei, Ali, Ismail *et al.*, 2011; Wei, Ali, Yoon *et al.*, 2011; Wei, Ali, Choon *et al.*, 2011). An intramolecular C29—H29A···O1 hydrogen bond (Table 1) forms an *S*(8) ring motif (Bernstein *et al.*, 1995). The naphthalene ring system (C2-C11) of dihydro-acenaphthylene group forms dihedral angles of 41.76 (6) and 42.17 (6)° with the benzene ring (C24-C29) of the bromophenyl group and the benzene ring (C15-C20) of the dihydro-indene group. The dihedral angle between the two benzene rings (C24-C29/C15-C20) is 83.92 (7)°.

The crystal packing is shown in Fig. 2. Weak intermolecular C4—H4A···O2^i^, C16—H16A···N1^ii^ and C26—H26A···O16^iii^ (Table 1) hydrogen bonds link molecules into a two-dimensional network parallel to *ac*-plane. The crystal structure is further stabilized by weak intermolecular C5—H5A···*Cg*1 (Table 1) interactions (*Cg*1 is the centroid of the C15–C20 ring).

### Experimental

A mixture of (*E*)-2-(4-bromobenzylidene)-2,3-dihydro-1*H*-indene-1- one (0.001 mol), acenaphthenequinone (0.001 mol) and sarcosine (0.002 mol) (1:1:2) were dissolved in methanol (10 ml) and refluxed for 4 h. After completion of the reaction as evident from TLC, the excess solvent was evaporated slowly and the product was separated and recrystallized from methanol to reveal the title compound as yellow crystals.

### Refinement

All H atoms were positioned geometrically [C–H = 0.95 and 1.00 Å] and refined using a riding model with *U*_iso_(H) = 1.2 and 1.5*U*_eq_(C). A rotating group model was applied to the methyl group. Four reflections 10 0 4, 7 15 12, -3 19 13 and -6 20 12 were omitted in the final refinement.

### Crystal data

**Table:**

C_30_H_22_BrNO_2_	*F*(000) = 1040
*M**_r_* = 508.40	*D*_x_ = 1.451 Mg m^−^^3^
Monoclinic, *P*2_1_/*c*	Mo *K*α radiation, λ = 0.71073 Å
Hall symbol: -P 2ybc	Cell parameters from 9857 reflections
*a* = 8.6638 (1) Å	θ = 2.5–33.4°
*b* = 19.9429 (2) Å	µ = 1.80 mm^−^^1^
*c* = 13.5225 (1) Å	*T* = 100 K
β = 94.937 (1)°	Block, yellow
*V* = 2327.77 (4) Å^3^	0.42 × 0.19 × 0.17 mm
*Z* = 4	

### Data collection

**Table:**

Bruker SMART APEXII CCD area-detector diffractometer	9279 independent reflections
Radiation source: fine-focus sealed tube	6879 reflections with *I* > 2σ(*I*)
Graphite monochromator	*R*_int_ = 0.034
φ and ω scans	θ_max_ = 33.9°, θ_min_ = 1.8°
Absorption correction: multi-scan (*SADABS*; Bruker, 2009)	*h* = −12→13
*T*_min_ = 0.522, *T*_max_ = 0.748	*k* = −29→31
31742 measured reflections	*l* = −19→21

### Refinement

**Table:**

Refinement on *F*^2^	Primary atom site location: structure-invariant direct methods
Least-squares matrix: full	Secondary atom site location: difference Fourier map
*R*[*F*^2^ > 2σ(*F*^2^)] = 0.036	Hydrogen site location: inferred from neighbouring sites
*wR*(*F*^2^) = 0.085	H-atom parameters constrained
*S* = 1.03	*w* = 1/[σ^2^(*F*_o_^2^) + (0.0334*P*)^2^ + 1.002*P*] where *P* = (*F*_o_^2^ + 2*F*_c_^2^)/3
9279 reflections	(Δ/σ)_max_ = 0.002
308 parameters	Δρ_max_ = 0.46 e Å^−^^3^
0 restraints	Δρ_min_ = −0.49 e Å^−^^3^

### Special details

**Table:**

Experimental. The crystal was placed in the cold stream of an Oxford Cryosystems Cobra open-flow nitrogen cryostat (Cosier & Glazer, 1986) operating at 100.0 (1) K.
Geometry. All e.s.d.'s (except the e.s.d. in the dihedral angle between two l.s. planes) are estimated using the full covariance matrix. The cell e.s.d.'s are taken into account individually in the estimation of e.s.d.'s in distances, angles and torsion angles; correlations between e.s.d.'s in cell parameters are only used when they are defined by crystal symmetry. An approximate (isotropic) treatment of cell e.s.d.'s is used for estimating e.s.d.'s involving l.s. planes.
Refinement. Refinement of *F*^2^ against ALL reflections. The weighted *R*-factor *wR* and goodness of fit *S* are based on *F*^2^, conventional *R*-factors *R* are based on *F*, with *F* set to zero for negative *F*^2^. The threshold expression of *F*^2^ > σ(*F*^2^) is used only for calculating *R*-factors(gt) *etc*. and is not relevant to the choice of reflections for refinement. *R*-factors based on *F*^2^ are statistically about twice as large as those based on *F*, and *R*- factors based on ALL data will be even larger.

### Fractional atomic coordinates and isotropic or equivalent isotropic displacement parameters (Å^2^)

**Table:**

	*x*	*y*	*z*	*U*_iso_*/*U*_eq_	
Br1	0.480071 (19)	0.521944 (7)	0.244244 (11)	0.02379 (5)	
O1	1.13667 (12)	0.37057 (5)	0.06701 (8)	0.0181 (2)	
O2	0.73424 (12)	0.23859 (5)	−0.19577 (7)	0.0196 (2)	
N1	1.02645 (13)	0.34177 (6)	−0.14796 (8)	0.0147 (2)	
C1	1.14407 (15)	0.31588 (7)	0.02741 (10)	0.0143 (2)	
C2	1.25652 (15)	0.26231 (7)	0.05489 (10)	0.0149 (2)	
C3	1.37067 (16)	0.25607 (7)	0.13200 (10)	0.0171 (3)	
H3A	1.3852	0.2895	0.1820	0.020*	
C4	1.46532 (17)	0.19825 (8)	0.13408 (11)	0.0199 (3)	
H4A	1.5426	0.1923	0.1876	0.024*	
C5	1.44836 (16)	0.15013 (7)	0.06027 (11)	0.0194 (3)	
H5A	1.5149	0.1122	0.0638	0.023*	
C6	1.33380 (16)	0.15627 (7)	−0.02048 (10)	0.0162 (3)	
C7	1.30942 (17)	0.11341 (7)	−0.10429 (11)	0.0187 (3)	
H7A	1.3706	0.0742	−0.1087	0.022*	
C8	1.19717 (17)	0.12873 (7)	−0.17900 (11)	0.0193 (3)	
H8A	1.1838	0.0999	−0.2350	0.023*	
C9	1.10026 (17)	0.18618 (7)	−0.17541 (10)	0.0168 (3)	
H9A	1.0238	0.1955	−0.2282	0.020*	
C10	1.11894 (16)	0.22784 (6)	−0.09453 (10)	0.0143 (2)	
C11	1.23720 (15)	0.21288 (7)	−0.01929 (10)	0.0147 (2)	
C12	1.03831 (15)	0.29248 (7)	−0.06780 (10)	0.0135 (2)	
C13	0.86380 (15)	0.28669 (6)	−0.04240 (10)	0.0129 (2)	
C14	0.84775 (16)	0.25846 (7)	0.06297 (10)	0.0145 (2)	
H14A	0.9399	0.2696	0.1084	0.017*	
H14B	0.7543	0.2766	0.0908	0.017*	
C15	0.83414 (15)	0.18367 (7)	0.04746 (10)	0.0142 (2)	
C16	0.85513 (16)	0.13264 (7)	0.11705 (11)	0.0182 (3)	
H16A	0.8831	0.1424	0.1850	0.022*	
C17	0.83410 (18)	0.06670 (8)	0.08463 (12)	0.0222 (3)	
H17A	0.8506	0.0311	0.1311	0.027*	
C18	0.78920 (18)	0.05166 (7)	−0.01488 (12)	0.0229 (3)	
H18A	0.7741	0.0063	−0.0350	0.027*	
C19	0.76666 (17)	0.10280 (7)	−0.08419 (11)	0.0198 (3)	
H19A	0.7348	0.0933	−0.1517	0.024*	
C20	0.79213 (16)	0.16845 (7)	−0.05171 (10)	0.0152 (2)	
C21	0.78710 (16)	0.23101 (7)	−0.11033 (10)	0.0150 (2)	
C22	0.79283 (15)	0.35678 (7)	−0.07174 (10)	0.0142 (2)	
H22A	0.7097	0.3489	−0.1265	0.017*	
C23	0.92408 (16)	0.39505 (7)	−0.11737 (10)	0.0157 (2)	
H23A	0.8830	0.4221	−0.1751	0.019*	
H23B	0.9794	0.4250	−0.0678	0.019*	
C24	0.71902 (15)	0.39472 (7)	0.00913 (10)	0.0148 (2)	
C25	0.55805 (16)	0.39731 (7)	0.00765 (11)	0.0182 (3)	
H25A	0.4969	0.3736	−0.0425	0.022*	
C26	0.48464 (17)	0.43384 (7)	0.07808 (12)	0.0210 (3)	
H26A	0.3748	0.4356	0.0758	0.025*	
C27	0.57526 (17)	0.46743 (7)	0.15114 (11)	0.0176 (3)	
C28	0.73546 (17)	0.46420 (7)	0.15679 (11)	0.0185 (3)	
H28A	0.7960	0.4864	0.2088	0.022*	
C29	0.80658 (16)	0.42809 (7)	0.08538 (11)	0.0181 (3)	
H29A	0.9164	0.4261	0.0886	0.022*	
C30	1.17364 (17)	0.36523 (7)	−0.18036 (11)	0.0200 (3)	
H30A	1.2386	0.3266	−0.1936	0.030*	
H30B	1.2269	0.3929	−0.1282	0.030*	
H30C	1.1542	0.3919	−0.2410	0.030*	

### Atomic displacement parameters (Å^2^)

**Table:**

	*U*^11^	*U*^22^	*U*^33^	*U*^12^	*U*^13^	*U*^23^
Br1	0.03158 (9)	0.01891 (7)	0.02255 (8)	0.00434 (6)	0.01203 (6)	0.00014 (6)
O1	0.0180 (5)	0.0160 (5)	0.0198 (5)	−0.0010 (4)	−0.0013 (4)	−0.0046 (4)
O2	0.0239 (5)	0.0195 (5)	0.0144 (5)	−0.0010 (4)	−0.0047 (4)	−0.0009 (4)
N1	0.0153 (5)	0.0139 (5)	0.0150 (5)	0.0004 (4)	0.0016 (4)	0.0022 (4)
C1	0.0133 (6)	0.0162 (6)	0.0134 (6)	−0.0021 (4)	0.0009 (5)	0.0006 (5)
C2	0.0141 (6)	0.0155 (6)	0.0151 (6)	−0.0003 (4)	0.0008 (5)	0.0011 (5)
C3	0.0164 (6)	0.0189 (6)	0.0156 (6)	−0.0007 (5)	−0.0010 (5)	0.0007 (5)
C4	0.0159 (6)	0.0247 (7)	0.0187 (7)	0.0014 (5)	−0.0017 (5)	0.0040 (6)
C5	0.0182 (6)	0.0193 (7)	0.0207 (7)	0.0039 (5)	0.0013 (5)	0.0044 (5)
C6	0.0166 (6)	0.0163 (6)	0.0160 (6)	0.0014 (5)	0.0024 (5)	0.0022 (5)
C7	0.0220 (7)	0.0150 (6)	0.0196 (7)	0.0039 (5)	0.0039 (5)	0.0002 (5)
C8	0.0249 (7)	0.0172 (6)	0.0160 (6)	0.0010 (5)	0.0030 (5)	−0.0018 (5)
C9	0.0202 (6)	0.0171 (6)	0.0129 (6)	0.0010 (5)	−0.0004 (5)	0.0001 (5)
C10	0.0155 (6)	0.0138 (6)	0.0136 (6)	−0.0005 (4)	0.0015 (5)	0.0008 (5)
C11	0.0145 (6)	0.0151 (6)	0.0146 (6)	−0.0005 (5)	0.0018 (5)	0.0013 (5)
C12	0.0134 (6)	0.0137 (6)	0.0131 (6)	−0.0004 (4)	−0.0009 (5)	−0.0005 (5)
C13	0.0139 (6)	0.0122 (5)	0.0122 (6)	−0.0004 (4)	−0.0005 (5)	−0.0004 (4)
C14	0.0172 (6)	0.0142 (6)	0.0118 (6)	−0.0015 (5)	−0.0002 (5)	−0.0001 (5)
C15	0.0137 (6)	0.0142 (6)	0.0146 (6)	−0.0012 (4)	0.0007 (5)	−0.0006 (5)
C16	0.0184 (6)	0.0188 (6)	0.0175 (6)	−0.0009 (5)	0.0008 (5)	0.0026 (5)
C17	0.0247 (7)	0.0170 (6)	0.0249 (7)	0.0011 (5)	0.0030 (6)	0.0049 (6)
C18	0.0281 (8)	0.0128 (6)	0.0282 (8)	−0.0017 (5)	0.0052 (6)	−0.0020 (6)
C19	0.0242 (7)	0.0164 (6)	0.0190 (7)	−0.0032 (5)	0.0033 (5)	−0.0037 (5)
C20	0.0163 (6)	0.0141 (6)	0.0150 (6)	−0.0020 (5)	0.0004 (5)	−0.0001 (5)
C21	0.0153 (6)	0.0147 (6)	0.0148 (6)	−0.0006 (4)	0.0005 (5)	−0.0016 (5)
C22	0.0156 (6)	0.0135 (6)	0.0130 (6)	0.0013 (4)	−0.0013 (5)	0.0000 (5)
C23	0.0193 (6)	0.0119 (6)	0.0157 (6)	0.0008 (5)	0.0006 (5)	0.0015 (5)
C24	0.0162 (6)	0.0120 (6)	0.0160 (6)	0.0009 (4)	0.0006 (5)	0.0016 (5)
C25	0.0171 (6)	0.0157 (6)	0.0214 (7)	−0.0022 (5)	−0.0003 (5)	−0.0016 (5)
C26	0.0171 (6)	0.0192 (7)	0.0273 (7)	−0.0010 (5)	0.0050 (6)	−0.0001 (6)
C27	0.0230 (7)	0.0130 (6)	0.0178 (6)	0.0017 (5)	0.0063 (5)	0.0022 (5)
C28	0.0225 (7)	0.0157 (6)	0.0171 (6)	0.0012 (5)	0.0006 (5)	−0.0013 (5)
C29	0.0173 (6)	0.0179 (6)	0.0189 (7)	0.0015 (5)	−0.0003 (5)	−0.0018 (5)
C30	0.0188 (6)	0.0200 (7)	0.0217 (7)	−0.0012 (5)	0.0044 (5)	0.0028 (6)

### Geometric parameters (Å, º)

**Table:**

Br1—C27	1.9043 (13)	C14—H14A	0.9900
O1—C1	1.2192 (16)	C14—H14B	0.9900
O2—C21	1.2151 (17)	C15—C16	1.3873 (19)
N1—C12	1.4604 (17)	C15—C20	1.3925 (19)
N1—C30	1.4606 (17)	C16—C17	1.393 (2)
N1—C23	1.4662 (17)	C16—H16A	0.9500
C1—C2	1.4718 (19)	C17—C18	1.401 (2)
C1—C12	1.5848 (19)	C17—H17A	0.9500
C2—C3	1.380 (2)	C18—C19	1.388 (2)
C2—C11	1.4061 (19)	C18—H18A	0.9500
C3—C4	1.414 (2)	C19—C20	1.3924 (19)
C3—H3A	0.9500	C19—H19A	0.9500
C4—C5	1.383 (2)	C20—C21	1.4769 (19)
C4—H4A	0.9500	C22—C24	1.5158 (18)
C5—C6	1.416 (2)	C22—C23	1.5418 (18)
C5—H5A	0.9500	C22—H22A	1.0000
C6—C11	1.4062 (19)	C23—H23A	0.9900
C6—C7	1.421 (2)	C23—H23B	0.9900
C7—C8	1.375 (2)	C24—C25	1.3941 (19)
C7—H7A	0.9500	C24—C29	1.395 (2)
C8—C9	1.424 (2)	C25—C26	1.395 (2)
C8—H8A	0.9500	C25—H25A	0.9500
C9—C10	1.3721 (19)	C26—C27	1.381 (2)
C9—H9A	0.9500	C26—H26A	0.9500
C10—C11	1.412 (2)	C27—C28	1.385 (2)
C10—C12	1.5244 (18)	C28—C29	1.3902 (19)
C12—C13	1.5831 (18)	C28—H28A	0.9500
C13—C14	1.5495 (18)	C29—H29A	0.9500
C13—C21	1.5533 (19)	C30—H30A	0.9800
C13—C22	1.5644 (18)	C30—H30B	0.9800
C14—C15	1.5093 (19)	C30—H30C	0.9800
			
C12—N1—C30	115.52 (11)	C20—C15—C14	111.10 (11)
C12—N1—C23	106.50 (10)	C15—C16—C17	118.23 (14)
C30—N1—C23	114.67 (11)	C15—C16—H16A	120.9
O1—C1—C2	126.66 (13)	C17—C16—H16A	120.9
O1—C1—C12	124.87 (12)	C16—C17—C18	121.49 (14)
C2—C1—C12	108.38 (11)	C16—C17—H17A	119.3
C3—C2—C11	120.63 (13)	C18—C17—H17A	119.3
C3—C2—C1	132.24 (13)	C19—C18—C17	120.20 (14)
C11—C2—C1	107.05 (12)	C19—C18—H18A	119.9
C2—C3—C4	117.74 (13)	C17—C18—H18A	119.9
C2—C3—H3A	121.1	C18—C19—C20	117.95 (14)
C4—C3—H3A	121.1	C18—C19—H19A	121.0
C5—C4—C3	121.74 (14)	C20—C19—H19A	121.0
C5—C4—H4A	119.1	C19—C20—C15	122.01 (13)
C3—C4—H4A	119.1	C19—C20—C21	128.94 (13)
C4—C5—C6	121.34 (13)	C15—C20—C21	109.02 (12)
C4—C5—H5A	119.3	O2—C21—C20	127.35 (13)
C6—C5—H5A	119.3	O2—C21—C13	125.48 (12)
C11—C6—C5	116.03 (13)	C20—C21—C13	107.17 (11)
C11—C6—C7	116.38 (13)	C24—C22—C23	114.40 (11)
C5—C6—C7	127.53 (13)	C24—C22—C13	116.36 (11)
C8—C7—C6	119.97 (13)	C23—C22—C13	104.86 (10)
C8—C7—H7A	120.0	C24—C22—H22A	106.9
C6—C7—H7A	120.0	C23—C22—H22A	106.9
C7—C8—C9	122.40 (13)	C13—C22—H22A	106.9
C7—C8—H8A	118.8	N1—C23—C22	103.83 (10)
C9—C8—H8A	118.8	N1—C23—H23A	111.0
C10—C9—C8	119.01 (13)	C22—C23—H23A	111.0
C10—C9—H9A	120.5	N1—C23—H23B	111.0
C8—C9—H9A	120.5	C22—C23—H23B	111.0
C9—C10—C11	118.35 (12)	H23A—C23—H23B	109.0
C9—C10—C12	132.54 (13)	C25—C24—C29	118.20 (12)
C11—C10—C12	109.07 (12)	C25—C24—C22	119.46 (13)
C2—C11—C6	122.45 (13)	C29—C24—C22	122.34 (12)
C2—C11—C10	113.59 (12)	C24—C25—C26	121.59 (14)
C6—C11—C10	123.85 (13)	C24—C25—H25A	119.2
N1—C12—C10	113.43 (10)	C26—C25—H25A	119.2
N1—C12—C13	101.69 (10)	C27—C26—C25	118.49 (13)
C10—C12—C13	117.09 (11)	C27—C26—H26A	120.8
N1—C12—C1	113.80 (11)	C25—C26—H26A	120.8
C10—C12—C1	101.36 (11)	C26—C27—C28	121.48 (13)
C13—C12—C1	109.96 (10)	C26—C27—Br1	119.86 (11)
C14—C13—C21	102.49 (10)	C28—C27—Br1	118.63 (11)
C14—C13—C22	119.57 (10)	C27—C28—C29	119.19 (14)
C21—C13—C22	110.48 (11)	C27—C28—H28A	120.4
C14—C13—C12	112.89 (11)	C29—C28—H28A	120.4
C21—C13—C12	106.97 (10)	C28—C29—C24	121.00 (13)
C22—C13—C12	103.99 (10)	C28—C29—H29A	119.5
C15—C14—C13	104.09 (10)	C24—C29—H29A	119.5
C15—C14—H14A	110.9	N1—C30—H30A	109.5
C13—C14—H14A	110.9	N1—C30—H30B	109.5
C15—C14—H14B	110.9	H30A—C30—H30B	109.5
C13—C14—H14B	110.9	N1—C30—H30C	109.5
H14A—C14—H14B	109.0	H30A—C30—H30C	109.5
C16—C15—C20	120.09 (13)	H30B—C30—H30C	109.5
C16—C15—C14	128.79 (12)		
			
O1—C1—C2—C3	5.4 (2)	C10—C12—C13—C22	−152.10 (11)
C12—C1—C2—C3	−178.06 (14)	C1—C12—C13—C22	92.99 (12)
O1—C1—C2—C11	−171.24 (13)	C21—C13—C14—C15	23.95 (13)
C12—C1—C2—C11	5.34 (14)	C22—C13—C14—C15	146.48 (11)
C11—C2—C3—C4	−0.7 (2)	C12—C13—C14—C15	−90.76 (12)
C1—C2—C3—C4	−176.92 (13)	C13—C14—C15—C16	162.48 (13)
C2—C3—C4—C5	1.9 (2)	C13—C14—C15—C20	−18.65 (14)
C3—C4—C5—C6	−0.7 (2)	C20—C15—C16—C17	0.4 (2)
C4—C5—C6—C11	−1.6 (2)	C14—C15—C16—C17	179.22 (13)
C4—C5—C6—C7	175.66 (14)	C15—C16—C17—C18	−1.6 (2)
C11—C6—C7—C8	0.64 (19)	C16—C17—C18—C19	0.9 (2)
C5—C6—C7—C8	−176.58 (14)	C17—C18—C19—C20	0.9 (2)
C6—C7—C8—C9	−1.2 (2)	C18—C19—C20—C15	−2.0 (2)
C7—C8—C9—C10	−0.1 (2)	C18—C19—C20—C21	175.43 (14)
C8—C9—C10—C11	1.82 (19)	C16—C15—C20—C19	1.4 (2)
C8—C9—C10—C12	179.18 (13)	C14—C15—C20—C19	−177.59 (12)
C3—C2—C11—C6	−1.7 (2)	C16—C15—C20—C21	−176.52 (12)
C1—C2—C11—C6	175.40 (12)	C14—C15—C20—C21	4.50 (15)
C3—C2—C11—C10	−177.99 (12)	C19—C20—C21—O2	13.6 (2)
C1—C2—C11—C10	−0.91 (15)	C15—C20—C21—O2	−168.64 (14)
C5—C6—C11—C2	2.75 (19)	C19—C20—C21—C13	−166.02 (14)
C7—C6—C11—C2	−174.79 (12)	C15—C20—C21—C13	11.70 (14)
C5—C6—C11—C10	178.69 (12)	C14—C13—C21—O2	158.12 (13)
C7—C6—C11—C10	1.15 (19)	C22—C13—C21—O2	29.63 (18)
C9—C10—C11—C2	173.85 (12)	C12—C13—C21—O2	−82.93 (16)
C12—C10—C11—C2	−4.09 (15)	C14—C13—C21—C20	−22.21 (13)
C9—C10—C11—C6	−2.4 (2)	C22—C13—C21—C20	−150.69 (11)
C12—C10—C11—C6	179.65 (12)	C12—C13—C21—C20	96.74 (12)
C30—N1—C12—C10	−61.01 (15)	C14—C13—C22—C24	3.30 (17)
C23—N1—C12—C10	170.39 (11)	C21—C13—C22—C24	121.81 (12)
C30—N1—C12—C13	172.37 (11)	C12—C13—C22—C24	−123.73 (12)
C23—N1—C12—C13	43.77 (13)	C14—C13—C22—C23	130.78 (12)
C30—N1—C12—C1	54.18 (15)	C21—C13—C22—C23	−110.71 (12)
C23—N1—C12—C1	−74.42 (13)	C12—C13—C22—C23	3.75 (13)
C9—C10—C12—N1	−48.4 (2)	C12—N1—C23—C22	−42.08 (13)
C11—C10—C12—N1	129.14 (12)	C30—N1—C23—C22	−171.17 (11)
C9—C10—C12—C13	69.61 (19)	C24—C22—C23—N1	150.47 (11)
C11—C10—C12—C13	−112.85 (13)	C13—C22—C23—N1	21.81 (13)
C9—C10—C12—C1	−170.79 (14)	C23—C22—C24—C25	133.34 (13)
C11—C10—C12—C1	6.75 (13)	C13—C22—C24—C25	−104.04 (15)
O1—C1—C12—N1	47.22 (17)	C23—C22—C24—C29	−46.03 (18)
C2—C1—C12—N1	−129.43 (11)	C13—C22—C24—C29	76.59 (16)
O1—C1—C12—C10	169.35 (12)	C29—C24—C25—C26	2.0 (2)
C2—C1—C12—C10	−7.30 (13)	C22—C24—C25—C26	−177.35 (13)
O1—C1—C12—C13	−66.10 (16)	C24—C25—C26—C27	−0.7 (2)
C2—C1—C12—C13	117.25 (11)	C25—C26—C27—C28	−1.5 (2)
N1—C12—C13—C14	−159.00 (10)	C25—C26—C27—Br1	176.49 (11)
C10—C12—C13—C14	76.81 (14)	C26—C27—C28—C29	2.1 (2)
C1—C12—C13—C14	−38.09 (14)	Br1—C27—C28—C29	−175.86 (11)
N1—C12—C13—C21	89.02 (12)	C27—C28—C29—C24	−0.6 (2)
C10—C12—C13—C21	−35.17 (15)	C25—C24—C29—C28	−1.4 (2)
C1—C12—C13—C21	−150.08 (11)	C22—C24—C29—C28	178.00 (13)
N1—C12—C13—C22	−27.92 (12)		

### Hydrogen-bond geometry (Å, º)

*Cg*1 is the centroid of the C15–C20 ring.

**Table:**

*D*—H···*A*	*D*—H	H···*A*	*D*···*A*	*D*—H···*A*
C29—H29*A*···O1	0.95	2.25	3.1110 (17)	151
C4—H4*A*···O2^i^	0.95	2.59	3.3743 (19)	140
C16—H16*A*···N1^ii^	0.95	2.50	3.4278 (19)	165
C26—H26*A*···O1^iii^	0.95	2.43	3.2597 (18)	146
C5—H5*A*···*Cg*1^iv^	0.95	2.71	3.3186 (15)	123

Symmetry codes: (i) *x*+1, −*y*+1/2, *z*+1/2; (ii) *x*, −*y*+1/2, *z*+1/2; (iii) *x*−1, *y*, *z*; (iv) *x*+1, *y*, *z*.
